# The Use of MSCs, iPSCs, and EVs in the Repair of Human MSK Tissues: Is Ultimate Success Dependent on Developing Excellent Implant Materials as Well as Creating an Optimal Environment for Implantation? What Is the Rationale for These Choices?

**DOI:** 10.3390/ijms26136250

**Published:** 2025-06-28

**Authors:** David A. Hart

**Affiliations:** Department of Surgery, Faculty of Kinesiology, and McCaig Institute for Bone & Joint Health, University of Calgary, Calgary, AB T2N 4N1, Canada; hartd@ucalgary.ca

**Keywords:** mesenchymal stem cells, tissue repair, tissue regeneration, mesenchymal stromal cells, extracellular vesicles, musculoskeletal tissues, pluripotent mesenchymal regulatory cells, induced pluripotent stem cells

## Abstract

It has been >35 years since the cells described as mesenchymal stem cells (MSCs) were reported to have multi-lineage potential, which opened the possibility that they could be used to repair injured or diseased musculoskeletal tissues. Since that time, similar cells have been isolated from many tissues, again raising expectations that they could be used to repair or regenerate many types of tissues. While some progress in using these cells, as well as induced pluripotent stem cells (iPSCs), to facilitate the repair of tissues has been achieved, an emerging body of literature would suggest that the cells in question facilitate repair via released extracellular vesicles (EVs) that contain a cargo of molecules which induce endogenous cells to do the actual repair. How the “stemness” of the cells is involved in such processes remains to be elucidated. While progress in the repair of compromised tissues has been obtained, from some perspectives, the progress has been challenging and successful translation to patients has been slow. In part, this has been due to considerable emphasis being placed on the cells or EVs, and not as much on the environments in which they are implanted. However, successful outcomes likely depend on both the development of optimized materials to be implanted and an environment that is conducive to success after implantation. This perspective article reviews some of the options regarding the implantable materials and the variables or factors that could impact the local environment’s suitability for success following implantation. In addition, attempts are made to reconcile the designation of endogenous cells labeled MSCs and their potential roles as regulators of tissue integrity in vivo.

## 1. Introduction

### 1.1. Purpose of the Review

With there being rapidly aging societies around the world, many individuals are suffering from a loss of the age-related integrity of many of their tissues, particularly those of the musculoskeletal system, which impact mobility and many aspects of daily living. Prominent among these conditions and diseases are osteoarthritis, intervertebral disc degeneration, osteoporosis, sarcopenia, and ligament and tendon injuries. For many of these conditions, effective interventions only treat the symptoms and do not lead to the return of tissue structural integrity.

The discovery of what were termed mesenchymal stem cells (MSCs) > 30 years ago [1,2,3] was a paradigm-shifting finding that initially offered the potential to repair and regenerate connective tissues. However, this initial euphoria led to considerable “hype” and the potential of these MSCs to meet these expectations was not realized to the extent that was suggested in the literature and by commercial entities. These limited and variable successes led to considerable research to better define the characteristics of the cells and their actual potential to repair and regenerate damaged connective tissues in vivo. This research has led to suggestions that, rather than “stem” cells, they are mesenchymal stromal cells (MSCs) or medicinal signaling cells (MSCs) [4,5], with the latter name reflecting on the fact that the cells in question may not repopulate damaged or diseased tissues but instead secrete mediators or release EVs that modulate the behavior of resident cells to facilitate repair. Thus, what were originally labeled as “stem” cells function in many tissues as “regulatory” cells [6]. Thus, much has been learned about the cells in question, in terms of both their strengths and limitations.

In contrast, studies to better define the state of the environment to which the MSCs and their products should be introduced, and whether to use cells or appropriately generated tissue engineered constructs containing MSCs or their products, have been less of a focus but are potentially equally important to the success of the interventions [7]. That is, putting optimally developed MSCs, their EVs with an optimal cargo content, or a specific tissue engineered construct into a suboptimal environment may very likely compromise the ultimate success of the intervention.

Therefore, this perspective article will present and discuss advances on both sides of the field of connective tissue repair and regeneration and attempt to address how to integrate the two research efforts to enhance the potential for success in the clinical translation of findings.

### 1.2. Background and History

The repair and regeneration of tissues, with the potential to prolong healthy life, has been a long-term goal of many clinician-scientists and researchers. Just replacing injured or diseased tissues can be accomplished by the well-controlled transplantation of allogenic donor tissues, but this approach is not without risks, and most individuals would prefer to use their own autologous tissues and cells, if possible, to repair and regenerate tissues.

#### 1.2.1. Mesenchymal Stem Cells

In lieu of tissue transplantation, another option for repair is the use of stem cells. Embryonic stems cells that arise during development are one option that could be explored, but there are ethical concerns with using human embryonic cells, and there is also a risk of such cells developing cancers such as teratomas [8,9].

Approximately 35 years ago, reports of MSCs came from Caplan’s lab [1,2] that offered another option to repair connective tissues. These cells were multipotent and could be differentiated into bone, cartilage, and adipocyte lineages using specific induction protocols. Such cells were found in nearly every tissue that was examined and could therefore be used in an autologous manner. The most used sources of MSCs for study in vitro or in preclinical models were from bone marrow [10], adipose tissue [11], and synovium [12,13], as well as cord blood [14], Wharton’s jelly [15], placenta [16], synovial fluid [17], amniotic fluid [18], and individual tissues. Of interest are the findings that those from bone marrow appear to prefer the osteogenic lineage when differentiated, while those from synovium appear to prefer the chondrogenic lineage reviewed in [6,12]; thus, MSC populations in different locations may serve specific functions in different environments.

MSCs are also heterogeneous at the cell level, as determined by limiting dilution analysis [17]. While some reports have raised the possibility that such heterogeneity is associated with in vitro culturing and passaging the MSCs in vitro, as discussed in [6,19,20], population studies would indicate that some heterogeneity is intrinsic to specific locations. Regarding autologous repair approaches, the potential for additional individual variation in the “quality” of the MSC population (i.e., via genetics and epigenetics) in any particular location could also impact the success of their use in repairing a damaged tissue [21,22].

In spite of a number of limitations [21,23] in the use of MSCs, reagents which are usually defined by their functional lineage differentiation phenotype and cell-surface antigen phenotype [24,25,26,27,28], some successes with connective tissue repair have been reported for conditions such as osteoarthritis, as reviewed in [29,30], and cartilage defects [31]. An example of one of these successes was the use of human synovium-derived MSCs in a tissue engineered construct (TEC) to effectively repair human knee cartilage defects [12,13,32,33,34]. In a small pilot study of five patients (the maximum allowed by the Japanese agency funding the study), the implantation of a TEC containing undifferentiated MSCs in a self-generated matrix led to the long-term (>5 years) repair of the damaged tissue [13,32,33,34]. The in vivo-generated repair tissue expressed collagen II and developed a superficial zone following implantation and, therefore, the implanted cells either differentiated into zone-specific chondrocytes or convinced endogenous cells to generate repair hyaline cartilage and replace the matrix of the TEC, which initially contained collagen I, with a collagen II-rich matrix. It is too soon to say that the injured cartilage was totally “regenerated” and, based on earlier results from studies in porcine models [35,36], the repair of hyaline cartilage may not have a completely regenerated surface. In spite of this potential limitation, the 5-year post-implantation findings are very positive regarding the ability of this intervention to prevent knee hyaline cartilage defects in humans from progressing to osteoarthritis. Based on the initial success of this TEC MSC pilot program, a larger multi-arm clinical trial is currently underway in Japan.

However, it should also be noted that the TEC implanted in these studies was mainly prepared using autologous MSC, so it is not possible to determine whether the implanted cells repopulated the repair tissue or facilitated repopulation by endogenous cells. However, this could be assessed further, as allogenic cells also appear to function well in this porcine model [37], and cells from females could be implanted into the cartilage defects of males and the cells in the repair tissue could be assessed by karyotyping. Thus, this approach of using synovium-derived MSCs is very effective in cartilage repair and demonstrates the successful translation from a relevant porcine model to humans [34]. With continued improvement in this technique, such as regulating the oxygen tension to enhance the cartilage-directed differentiation of the MSC [38], current successes may be further optimized.

In addition to cartilage repair, this approach of using a TEC and synovium-derived MSCs has also shown some success in other applications in relevant preclinical models. These include the repair of osteochondral defects in a rabbit model [31], as well as the repair of meniscal defects in both rabbit [39] and porcine [40] preclinical models. As meniscal injuries are common and their repair is critical to preventing the development of osteoarthritis, the translation of preclinical findings to human conditions should also be a priority [41]. Therefore, the continued optimization of the use of TEC in relevant preclinical models may lead to additional clinical trials in the near future.

#### 1.2.2. Extracellular Vesicles

While much research continues to investigate the use of MSCs in the repair of musculoskeletal tissues and others, it was noted that many cells, including MSCs, release small EVs during their culture, as discussed in [42,43,44,45], and these vesicles contain a variety of bioactive molecules including proteins, miRNA, mRNA, and lipids [46]. The content or “cargo” of EVs can vary depending on the differentiation status of the MSC, the source of the cells, as discussed in [47], the age [48,49] and sex [50,51] of the donor, and the species of the donor, as discussed in [52]. The functioning of EVs may be the mechanism by which MSCs are able to facilitate repair via modifying the activity of endogenous cells in the target site and their ability to respond appropriately. Thus, EVs could be released by appropriate MSCs, recognized by endogenous cells via membrane-associated recognition systems, as discussed in [20], and then taken up by these cells to facilitate cell activation/modification that leads to an enhanced repair capability.

Since 2000, the field of EV generation, characterization, and function has expanded dramatically, with international journals dedicated to publishing relevant articles (i.e., *Journal of Extracellular Vesicles*) and organized societies formulating criteria for their study, definitions, guidelines, position papers, and characterization (i.e., International Society for Extracellular Vesicles; http://www.isev.org, accessed on 24 June 2025). Such efforts have greatly advanced the field as, early on, the names used to describe these vesicles were varied (i.e., exosomes, different names for vesicles of different sizes, extracellular vesicles) and attempts to standardize names and characteristics have allowed for more clarity and a better ability to compare the results of different studies.

As with the early days of MSCs, the field of EVs has been complicated by the conditions of generation (i.e., cells they are derived from, cargo content variation, and conditions of generation—i.e., presence of serum or serum-free culture) discussed in [53,54,55]. Regarding the latter point of potential serum effects, it appears that the generation of serum, such as the widely used fetal bovine serum (FBS), leads to contamination by EVs present in the serum, as well as small-sized particulates that may be generated by the actual coagulation process [55]. Not only would the presence of such bovine-associated EVs complicate the study of MSC-derived EVs, serum-associated EVs and particulates could also interfere with the activity of MSC-derived EVs generated in the presence of serum [55]. Therefore, if a serum source is required in a study of EVs, it should be centrifuged at >100,000× *g* for an extended period of time to eliminate these confounding elements.

In spite of the emerging nature of EV applications, preparations of EVs have been investigated for their efficacy in treatments for osteoarthritis [56,57,58], intravertebral disc degeneration [59], tendon injury [60], ligament healing [61], bone integrity and repair [62,63,64], meniscal repair [65,66,67], chronic wounds [68], and muscle [69]. Thus, EVs may be useful for mediating connective tissue repair in a variety of circumstances and their cargo of miRNA may play important roles in the repair of connective tissues such as bone and cartilage [70], the intervertebral disc [71], and the meniscus [72].

While EVs may be an important mechanism for the functioning of MSCs, offer the advantage of having a minimal potential to cause cancer, and have no nucleus, they do have the limitation of a short half-life when applied in vivo. That is, if effective repair requires prolonged exposure to EV, having the continuous generation of EVs via implanted MSCs rather than the repeated injection of EVs may be the most effective strategy to optimize repair. At some point, the implanted cells would no longer be needed and then would perhaps die by a mechanism that would not induce subsequent inflammation. This concept is exemplified by a recent study by Nakagawa et al. [73] that used human donor cells in a nude rat model, where implantation of the human constructs led to the effective repair of osteochondral defects but human cells were no longer present in the mature repair tissue.

#### 1.2.3. Induced Pluripotent Stem Cells

While MSCs/PMRCs have long been the focus of intense investigation regarding their “stem cell” or multi- or pluripotency potential to repair tissues of the MSK system, a second option regarding pluripotency arose from the work of Yamanaka’s laboratory, which described the ability to “reprogram” adult somatic cells in vitro using a cocktail of four factors (Oct3/4, Sox2, c-Myc, and Klf4) [74,75]. These cells, called induced pluripotent stem cells (iPSCs), offer potential for tissue repair, but the original description using c-Myc raised concern regarding the potential to result in tumors and led to attempts to generate the cells without c-Myc [76]. Since 2006, many investigations have characterized the generation and use of iPSCs in a variety of repair/regeneration situations, as reviewed in [77,78,79,80], and improvements in the protocols for generating iPSCs are discussed in [81,82,83].

Included in these studies on iPSCs are several which are focused on different tissues of the MSK system, as reviewed in [84], such as tendons [85,86,87,88], cartilage [89,90,91,92,93,94,95], intervertebral discs [96,97], bone [98,99,100], and ligament [101]. Most studies are in vitro or in animals, including mini pigs [95], where iPSCs and MSCs were compared, horses [102,103], goats [104], and companion animals [105]. While a number of studies have used iPSCs in large animal models, there is a paucity of information regarding clinical trials in patients that used MSK applications. However, some in the dental field have been reported, as reviewed in [106]. There is a need for more clinical trials in this area, as discussed in [107], and some countries such as Japan have a national program to foster and advance the clinical applications of different types of stem cell therapy for a variety of conditions, as discussed in [108].

Similar to natural MSCs, iPSCs can also generate EVs, and these EVs have been studied for their cargo content and characterized for their potential application in tissue repair [43,109,110,111]. When EVs from MSCs and iPSCs were compared, considerable variation in their cargo content was observed [112], as well as lineage-specific differences [113]. Thus, for specific applications regarding connective tissue repair, generating optimized EV cargo content will be required to enhance the potential for success of this method. However, continued assessment and investigation may identify some commonalities, such as defined miRNAs that are essential, as discussed in [111]. Thus, both MSCs and iPSCs may be used for specific applications to repair MSK tissues, and their respective EVs may also be used to better characterize how the implanted cells impact endogenous cells to facilitate repair/regeneration.

In summary, there are several different options for using multipotent cells or EVs derived from such cells to repair damaged connective tissues of the musculoskeletal system, with each option having advantages and limitations (Table 1). While considerable progress in understanding both the cell and tissue biology of these options and their potential applications to improve the repair of damaged or diseased tissues has been made, much of the research remains in vitro or in preclinical models. Optimization of the success regarding outcomes will depend on factors such as the source/origin and quality of the cells or EVs to be implanted, as well as the biological site of implantation, the state of the environment (i.e., inflammation; anabolic or catabolic) that the cells or EVs will be implanted in, the nature of the scaffold use to keep the materials at the site of implantation, and the biomechanical environment [114].

## 2. Do Names Matter? -Scientifically and Clinically, Yes

As outlined in Section 1, the field(s) of connective tissue repair and “regeneration” use what were initially labelled MSCs (mesenchymal stem cells) [1,2] and then emerged as MSCs (mesenchymal stromal cells) followed by MSCs (medicinal signaling cells) [4,5] as the field matured and more insights were illuminated, particularly as the advent of a body of knowledge regarding EVs developed. Thus, over time, less emphasis was placed on the “stem” aspects and more on the regulatory aspects of the cells, particularly with regard to EVs. Therefore, names do matter as they should be accurate, reflect the cells’ function, and can likely influence expectations by both researchers and patients.

While the abbreviation for the cells in question, MSC, has been retained through their history, their role in tissue repair has evolved and, thus, the retention of MSC as a name is also somewhat confusing for the literature and, potentially, patients who are targeted by commercial companies offering “stem cells” in the treatment of connective tissue diseases such as osteoarthritis, or even diseases and conditions of other biological systems (i.e., brain, heart, liver, kidneys). Thus, they could be called pluripotent mesenchymal regulatory cells (PMRCs), a name which recognizes their multi-lineage potential, their mesenchymal origins, and their regulatory function [6], or, more appropriately, multipotent mesenchymal regulatory cells (MMRCs). Such names also do not presuppose that their primary role is related to tissue repair and regeneration, which the field appears to have accepted without considering other roles. Their regulatory role, the finding that these cells are located as pericytes in nearly all tissues [3,115], and their decline in numbers and perhaps function with age (decreasing at a time in the lifespan when they might be more needed for tissue repair functions) may indicate that they have other functions earlier in life such as during times of rapid and integrated growth and maturation of tissues and organ systems [115]. The ability of these cells, initially labeled MSCs, to enhance the repair of damaged tissues could also play important functions in the coordinated and integrated growth associated with the period of time from birth to puberty and then from puberty to skeletal maturity [115]. The function of these cells in the growth and maturation following puberty may mean that they are further regulated by sex hormones to some degree. This could explain some of the reported sex-specific differences in EVs derived from these and other cells [116,117]. That this concept has validity is shown by the results of the studies of Nakagawa et al. [73], who showed that, in a xenogeneic model, the implanted cells disappeared and were replaced by endogenous cells in the repair tissue. Such findings also indicate that the mediators of the effect(s) of the implanted cells (i.e., EVs and/or secreted molecules) are not species-specific, as human cells were implanted into an immune compromised rat model. Consistent with the concept that MSCs/MMRCs and iPSCs function mainly via using EVs to facilitate the repair of damaged tissues of the MSK system (discussed in Section 1.2.2), the paracrine functions of these cells dominate their function in vivo rather than their “stem cell” characteristics. If that is the case, why do MSCs retain their “stem cell” characteristics when isolated from a variety of tissues? Further, there are two other important questions: (1) is “stemness” necessary to develop specific lineages and subsequently specific cargos in the EVs; and (2) is “stemness” related to other, as yet unknown, functions of the cells, as indicated by them maintaining tissue integrity when they are subjected to environmental and metabolic stresses? In addition, while there are some characteristics which differ between MSCs derived from different tissues [118], there could also be different subsets of cells with “stemness” or multipotency within a tissue, and they may have different functions. This topic should be a focus of future studies as even MSCs that freshly isolated from tissues appear to be heterogeneous [17].

While the cells currently called MSCs by nearly all of the field may have other functions, that does not detract from their “stem cell” capabilities, as they have demonstrated the potential to repair tissues damaged by disease and/or injury. However, if they have multiple roles to play in health and disease, one should not lose that perspective, and in that regard, names likely matter to those active in the field and those looking to the field for research and clinical application guidance. From this perspective, the environment into which such cells are implanted will likely influence the ability of such cells to perform specific functions. Thus, attempting to function in an inflammatory environment may be critical for specific response patterns, as discussed in [102].

## 3. Does the In Vivo Environment Matter? Factors Potentially Contributing to the Implantation Environment

Following injury to a connective tissue such as those within joints, tendons, skin, muscles, or oral cavities, the host usually responds to the insult with an acute inflammatory response. This response is designed to remove damaged tissue components, thwart any infectious agents, and initiate a rapid fibrotic response. In tissues such as skin, this leads to scar formation and protection of the host via closing the breach in this protective layer. However, a similar breach in the mucosal surface of the oral cavity heals with an outcome closer to regeneration [119,120].

While this outcome mediated by inflammatory responses is protective for skin, for connective tissues that require structural features to function optimally in a biomechanically active environment, such as ligaments, tendons, articular cartilage, and menisci, this “protective” inflammatory response leads to mechanically inferior outcomes in nearly all cases, as discussed in [114]. This concept can be observed during development, as some injuries to the human fetus can heal via regeneration (i.e., scarless wound healing) early during gestation, and this response then converts to a scar formation response later in development, as discussed in [121,122,123]. This conversion is associated with several factors including the development of an inflammatory response, as discussed in [124,125,126]. It has also been demonstrated in some adult preclinical models where treatment immediately after injury with anti-inflammatory agents (i.e., glucocorticoids) prevents the development of osteoarthritis-like disease in the knee. This has been reported for multiple preclinical models including rabbits [127,128,129,130], sheep [131], and pigs [132], and appears to be a general phenomenon and not species-specific. However, evidence for large clinical trials in patients could not be found. When interventions such as corticosteroids are given, the circumstances may impact the outcomes [133]. Caution in this regard is likely warranted as high doses of steroids can influence intraarticular MSC function [134].

Regarding the role of acute inflammatory processes in the environment being considered for an MSC/iPSC/EV intervention, additional points need to be raised and discussed. First, an acute inflammatory response can evolve into a chronic inflammatory response if the initial insult persists, as discussed in [135,136,137], and a chronic inflammatory state can be challenging to control. As a chronic inflammatory state has features which differ from an acute inflammatory reaction, it can be difficult to regulate and interfere with chronic conditions such as osteoarthritis [138,139,140,141], which is often a target of stem/stromal cell therapies [142]. Thus, a non-healing chronic wound in a person with diabetes, such as foot ulcers [143,144] or a tendon rupture [145], may be an environment that could compromise the success of cellular and/or EV therapy. Furthermore, it has been reported that iPSCs can be influenced by inflammatory cytokines [102], so their function could be impacted by the environment.

A second point is that genetic or stochastic variables that occur shortly after an acute injury to a connective tissue can influence long term outcomes even without an intervention. Using rupture of the human Achilles tendon as an example, it has been shown recently using proteomic approaches that that the analysis of tissue samples and fluids taken days (time of surgery) or weeks (2 weeks post-surgery), respectively, after the injury can identify biomarkers that can predict excellent or poor outcomes at 1-year post-injury, as reviewed in [146,147,148,149,150,151]. Several of these biomarkers represent elements of inflammation and immune-mediated events. Defining what constitutes an environment that leads to a favorable outcome versus an unfavorable outcome and developing interventions to enhance poor endogenous environments would be critical for enhancing the potential success of any MSC/iPSC/EV interventions in patients with such environments following an injury (i.e., complete rupture of a tendon) or chronic inflammation of the tendon (i.e., chronic tendinopathy).

A third point relates to the influence of co-morbidities on the environment following an injury or during a chronic connective tissue disease. Thus, conditions like diabetes [152] and kidney disease [153] can contribute to dysfunctional healing and repair processes. In patients, diabetes can contribute to the development of chronic wound risk [152,154,155], and in rat models of tendon healing, the induction of diabetes can contribute to compromised healing [145]. Dysregulated glucose control in diabetic states can also lead to altered extracellular matrices due to the formation of advanced glycation end (AGE) products [156,157]. Such alterations can subsequently lead to interactions with macrophages that express the receptor for AGE (RAGE), contributing to an inflammatory environment [158]. An environment with compromised natural healing may also contribute to a lack of success with MSC/iPSC/EV interventions as well.

In addition to co-morbidities, self-inflicted conditions resulting from smoking can lead to both alterations in endogenous MSC cells [159,160] and alterations to long-term connective tissue repair outcomes [161,162], although there are some mixed results in this area, as reviewed in [163]. In part, the effect of smoking may include effects on the vascular system [164]. In addition to effects on MSCs, smoking may also impact the function of iPSCs [165,166,167]. Therefore, smoking and vaping should be avoided to remove deleterious effects on both the multipotent cells and the environment.

As many of these co-morbidities appear with aging, in which older patients may require more interventions to repair injured and/or diseased tissues, the local and systemic environments of the patient likely need to be addressed if they can compromise the effectiveness of MSC/iPSC/EV-based interventions. Thus, a fourth point relates to healing environments that are not compromised by co-morbidities but are instead influenced by “aging” processes. This consideration of the environment is complicated by the disconnect between biological and chronological aging, as discussed in [168,169,170]. However, for some tissues such as tendons and ligaments, the stiffness of the tissues changes with age, possibly due to alterations in the expression of the lubricant PRG4 [171,172,173]. There can also be increases in collagen cross-linking with age [174,175], another alteration which influences the mechanical properties of the tissue. Furthermore, there also appears to be a decline in the number of MSCs in tissues with age, and if these cells are involved in responses to MSC/EV interventions for connective tissue repair/regeneration, then, with increasing age, there would be fewer cells to respond, and these cells might be functionally compromised [176].

A fifth point regarding the environment relates to sex differences and also the fact that, for females, the hormonal environment is not constant. Furthermore, the hormonal environment can influence the inflammatory environment [177,178,179,180,181]; thus, a critical feature of the environment that any MSC/iPSC/EV-based intervention would be implanted in in vivo is variable across the lifespan in females (i.e., puberty, skeletal maturity, menopause). Furthermore, after puberty and before menopause, the hormonal environment would be variable depending on menstrual cycles and pregnancy. After menopause, the environment would be deficient of estrogen and progesterone but still have testosterone present. This, of course, can be altered by taking HRT and HRT-like regimens, but some secondary sequalae of menopause may still be altered.

From this discussion, it is clear that “one size does not fit all” when it comes to the optimal environment for MSCs/iPSCs/EVs alone or in engineered constructs to be implanted in (summarized in Table 2). As such, the expectations regarding the success of the outcomes of such implantations will have to be clearly managed. That is, can such implants be used to enhance outcomes in environments compromised by co-morbidities, inflammatory conditions, age or genetics, or is tissue regeneration still the goal? The former is likely a more pragmatic and achievable goal, while the latter is a worthwhile pursuit, but is likely going to be a long-term effort that will have to be tailored for the best MSCs/iPSCs/EVs for unique subsets of environments. While the focus of this discussion has been connective tissues of the MSK system, many of the considerations may also apply to other organ systems such as degenerative neurological conditions, renal diseases, and pulmonary conditions.

It should also be pointed out that the presence of co-morbidities and the medications used to control such complications, as well as aging factors, may not only influence the environment that MSCs/iPSCs/EVs are implanted into but also the quality of the materials that are implanted if autologous cells/EVs are generated for re-introduction. Thus, in some circumstances, the use of minimally immunogenic allogenic materials should be considered.

## 4. How Can MSCs, iPSCs, and EVs Be Optimized and Matched with a Suitable Environment?

From the above discussions, it is evident that optimizing both the materials to be implanted to facilitate damaged tissue repair and the environment into which they will be implanted are likely both critical for the expected level of success regarding the outcomes. Furthermore, consideration should be given to the integration of their development rather than their independent evaluation.

For this approach to succeed, the initial target should likely be a specific patient subset, with definable criteria being used. For acute situations, such as the trauma-induced development of a cartilage defect, the rupture of a tendon such as an Achilles tendon (AT), or severing a flexor tendon of the hand, this would likely require a rapid response protocol using allogenic MSCs/iPSCs or EVs prepared for the situation.

For the rupture of an AT, a rapid biomarker assessment protocol to determine whether the patient was a “good” or poor” outcome candidate [146,147,148] or at risk to develop a DVT [149], as well as the sex of the patient, would lead to the implantation of a suitable pre-prepared delivery system since the window of opportunity is short. As EVs and MSCs are reported to be of low immunogenicity, as discussed in [182,183,184], they could theoretically be used without fear of rejection until they were no longer required to facilitate repair by endogenous cells. As demonstrated by the study by Nakagawa et al. [73], even in a rat model where immune rejection was not an issue, the implanted human MSCs and iPSCs disappeared from the mature repair tissue. In the case of a ruptured flexor tendon, one would likely have to consider the repair of the tendon proper, as well as the sheath [185,186,187,188,189]. In this situation, not only is tissue repair an issue, but the inhibition of adhesion formation between the tendon and sheath [188,189] is also required. This latter target may require the addition of a further intervention such as the use of a molecule such as a lactoferrin peptide to induce PRG4, a boundary lubricant that can minimize adhesion [190,191]. These issues are likely important to resolve as MSCs have been used for flexor tendon repair with variable results, as reviewed in [192].

In the case of a chronic condition such as osteoarthritis, where hyaline cartilage needs to be repaired/regenerated, a somewhat different approach likely needs to be considered. First, the inflammation needs to be controlled. Second, there should be sufficient endogenous tissue and cells available to allow the implanted cells/materials to facilitate repair. Therefore, repair should be considered early rather than later in the disease progression when little endogenous tissue remains. This approach conceptually goes against much of current medical practice, which generally endorses a more conservative approach until the disease progresses, a timeline that may diminish the opportunity for cellular therapy success. A limitation of addressing tissue repair in osteoarthritis of the knee, for example, is that one may not know when the condition actually started, as diagnosis is usually dependent on the appearance of symptoms; secondarily, the term osteoarthritis is an umbrella term for multiple causations [193]. One subset includes trauma to structures such as the anterior cruciate ligament that can affect the biomechanical functioning of the knee as an organ system, a situation that may put excessive loading on the repair tissue and thus lead to the reoccurrence of osteoarthritis due to degeneration of the repair tissue.

The induction of a cartilage defect in the femoral condyle or tibial plateau presents an interesting scenario for the use of MSCs in a TEC to repair this defect in patients [12,13,32,33]. It can be uniquely addressed, as it is known when the defect occurred, and one can derive effective MSCs populations and subsequently EVs from them, elements that can be used to generate good outcomes based on the results of pilot studies [13,32,33]. One could also attempt to regulate the inflammatory environment via the controlled use of corticosteroids [127,128,129,130,131,132]. Finally, one can debride the edges of the cartilage defect to generate a suitable implant site. As well, the TEC used in such studies adheres to the defect site without the need for suturing, which is also an advantage. Thus, one could generate a TEC for use in such circumstances using autologous cells or have a pre-prepared TEC that incorporates appropriate allogenic cells. However, as a cartilage defect site can progress to overt osteoarthritis over time, it is still not clear as to when such a site may convert from an “acute” site to a “chronic” site and require a modified approach to facilitate optimized repair.

While EVs may need to be optimized for specific applications, MSCs or advanced iPSCs will likely still be needed for implantation in vivo for many applications if EVs are the vehicle for the cellular effects; thus, the situation may require the chronic generation of EVs from the cells to optimize long-term tissue repair. Furthermore, the “stem cell” properties of MSCs and iPSCs may be very useful in the treatment of diseases and conditions where the loss of endogenous matrix and cells is extensive, but this type of application may require unique scaffolds that mimic the natural ECM to encourage the appropriate differentiation of the implanted cells.

## 5. The Way Forward: What Is Achievable?

One of the main questions that arises from the discussion in Section 4 is whether it is technically feasible to both generate an optimal cellular component to facilitate the effective repair of damaged connective tissue and to optimize the in vivo environment to enhance outcomes to meet expectations. Failing that set of circumstances, what is the best compromise to realistically offer a fairly long-term solution that improves the quality of life for the affected patients? It is likely that not all of the variables outlined in Section 4 exert equal influence on the outcomes; therefore, the understanding of some will have a higher priority than that of others. In this regard, some of the variables that can be defined (i.e., biological age, sex, obesity, co-morbidities, history, inflammatory biomarkers) may be investigated, but the role of others such as genetic variability may prove to be more challenging at some levels but not others. For example, if a “good” versus “poor” outcome after a rupture of the AT [146,147,148,149,150,151] has a genetic basis, then the genetic variation could be defined and addressed with tailored interventions. However, if the basis for different outcomes is primarily stochastic, then finding a solution may be more challenging but still addressable on an individual basis.

It is clear that after >35 years since their discovery, the expectations surrounding MSCs (mesenchymal stem cells), as originally described for connective tissues as well as for the cells in the brain—to address serious brain conditions such as Alzheimer’s, Parkinson’s, and other degenerative conditions—have perhaps not been met regarding their use to repair damaged or diseased tissues.

While progress has been and is being made in the use of MSCs, iPSCs, and EVs in several connective tissue conditions, many challenges have been encountered which have contributed to variable results when attempts to repair and regenerate human connective tissues have been undertaken. However, advances have been made in using MSCs in areas such as cartilage defect repair [32,33,34,194], knee osteoarthritis, as reviewed in [194], IVD degeneration, as reviewed in [195], and bone healing, as reviewed in [196], as well as in using iPSCs in primate models [197] and human iPSCs into rat models [73,198]. In human clinical trials, variability in outcomes has been noted for some of the conditions that were investigated, so new understanding is required, particularly with regard to the host environment. However, with regard to this latter issue, some advances have also been made using non-invasive techniques such as the MRI assessment of degenerated IVD to reveal alterations in oxygen and glucose levels that could affect the outcomes after the implantation of MSCs/iPSCs/EVs [199,200].

Thus, a serious analysis of what has been learned during those 35 years, as well as a better understanding of why better progress has not been made, needs to be carried out. A major part of the problem with both connective tissue conditions and diseases, as well as brain conditions, is that we do not understand the basic disease mechanisms and are thus mainly treating symptoms rather than disrupting disease processes and regenerating damaged tissues that retain optimal function.

One goal going forward should be to ask more questions regarding what these cells labeled MSCs (or MMRCs) are actually doing in the various tissue they reside in normally. They do exhibit multipotency, and they likely contribute an endogenous role via appropriate paracrine signaling, which leads to the micro-repair of some differentiated cells in specific tissues. The finding that MSCs in bone marrow appear to prefer the osteogenic lineage while knee synovial MSCs prefer the chondrogenic lineage contributes to such conclusions, indicating location-specific preferences. The extensive heterogeneity of MSCs isolated from the same specific location is still a topic that needs explanation, as discussed in [17,201]. Some of the heterogeneity in MSC populations has been proposed to be an artifact of in vitro culturing, as discussed in [6,19,20], and if this is the case, why does it arise (i.e., inherent instability, in vitro culture conditions)?

Given some of the limitations associated with MSCs, should the field refocus to more extensively investigate the use of iPSCs, which can be generated under defined conditions, or are iPSCs just another approach to developing more effective EVs? While there is currently cancer risks associated with iPSCs generated using c-Myc as one of the factors [202,203,204], continual improvements in the technology may lead to a more risk-free cell population [83,205,206]. Thus, a well-characterized set of iPSCs, to be used either in an autologous or allogenic manner, may be a valuable alternative to the use of endogenous MSCs. However, both MSCs and iPSCs may offer advantages for specific applications, such as the generation of EVs with specific cargos. Thus, both cell types may have unique advantages and should continue to be investigated.

A particular focus should continue to be placed on the emerging role of EVs in facilitating the repair of MSKs and other tissues. Excellent progress has been made in characterizing the efficacy of these methods and some of this progress has been detailed in recent reviews by Nallakumarasamy et al. [207], Teo et al. [208], Liao et al. [209], and Kasula et al. [210]. As reviewed by Zhang et al. [211], EVs appear to influence the activities of several cell types during skin wound healing, including endogenous fibroblasts and immune cells involved in inflammatory processes. Thus, EVs from donor cells can interact with multiple cell types. Whether they use different membrane molecules to interact with different cell types or some common recognition system remains to be determined, and as reviewed by Hart [20], there are several options that are known. It may be possible to also modify their targeting, as described by Antes et al. [212]. Further characterization of their targeting and mechanisms of action could lead to engineered EVs that could target specific cells and serve as drug delivery systems, as discussed by Rodriguz and Vader [213], Abid et al. [214], and Liu et al. [215]. The continued evolution of such studies could lead to artificially generated EVs with specific targeting molecules, cargo contents, and applications for specific MSK conditions, as reviewed by Rosso and Cauda [216]. Such studies could also offer advantages to overcome any local environmental variables that inhibit “natural” EV function (i.e., proteinases, glycosidases, lipases, pH). Thus, the continued development of materials to be implanted or injected is an on-going research effort, and efforts to optimize their function and specificity should be performed in parallel with other activities related to the environment that they will be implanted in.

Whichever choice of cells and derived EVs is pursued, and likely all should be pursued at this point, the use of optimal preclinical models should be entertained. It is likely that this should involve large animal models rather than rodents. Considerable investigation of the use of MSCs in rodent models has been accomplished over the past 30+ years and it has been found that they do offer the advantage of restricted genetic heterogeneity; further, the ability to knock out genes and the cost of using large numbers is manageable. However, the success rate of translating the findings from inbred rodents to humans is not high, which is likely due to the considerable heterogeneity in human populations. Thus, the strength of the inbred mouse models, genetic restriction, may be a weakness for translation to a heterogeneous population.

Therefore, to enhance the likelihood of successful translation of the findings to human populations, the focus should perhaps be on large animal models that reflect the human population more accurately. For example, different breeds of domestic pigs exist which provide some genetic heterogeneity, and the genomes of these breeds have been sequenced [217,218,219], as well as that of miniature swine [220]. Their physiology has features that are similar in some aspects to humans, as discussed in [220], some of their joints are similar in size to humans, as discussed in [221,222], their weight and metabolism can be modified by diet, they can be manipulated at the genetic level if required by selective breeding, and they breed well, yielding large numbers of offspring per litter. However, they are still quadrupeds and are costly to maintain. As an example of successful use, Nakamura’s group used porcine models to validate the use of TEC containing synovial MSCs, as reviewed in [34,35,37], prior to the effective translation of the technology to human patients with cartilage defects [13,32,33]. Thus, the use of porcine models has proven valuable in this circumstance and may also prove to be valuable in other well-designed projects that involve the use of MSCs/iPSCs/EVs to address human tissue repair applications.

Building on the findings from the Nakamura group and others using porcine models, one could further investigate the ability to regulate the inflammatory response following an injury to a joint, tendon, ligament, or muscle or in the context of a co-morbidity such as diabetes. Furthermore, while the translation of the cartilage defect model using a TEC was successful [13,32,33], one could also investigate the effect of the time post-injury on the ability to obtain successful repair. As the time post-cartilage defect injury leads to the induction of an osteoarthritis-like condition in both animals [223,224] and humans [225,226], such studies may be very informative for assessing future translation potential. For example, in the porcine model used by Nakamura’s group to repair cartilage defects, instead of repairing the defects immediately, one could initiate defects and then attempt repair with a TEC using synovial MSCs at increasing times post-initiation to assess when and how the local environment becomes altered and poses risk for a decline in the success of the implantation. Learning how to improve the in vivo environment may then lead to translational interventions to enhance implantation in patients with early OA.

In addition to porcine models, other large models to be considered include sheep [227], goats [104], and horses [224]. The use of cats and dogs has fallen out of favor in many places due to reluctance to use companion animals, and the use of non-human primates is not only ethically challenging but also very costly but may be feasible at some specialized centers. In spite of these limitations, some chondrodystrophic dogs can be good models for connective tissue disease studies and provide valuable insights into disease processes, such as in the studies of Thompson et al. [228]. In addition, companion animals such as dogs and cats may also be the target of clinical applications for tissue repair, so some use of them in research may be warranted and necessary. Similarly, horses are both companion animals and used for racing so should also likely be used in some studies.

Aside from the use of in vivo models to gain further insights into the role of MSCs, some in vitro studies using cells, organoids, or connective tissue explants may provide additional information regarding the function of these cells. However, as nearly all of the connective tissues involved in the in vivo conditions function in mechanically active environments, care should be taken to perform in vitro studies in mechanically active environments to avoid forming conclusions that are not relevant when cells are mechanically loaded. Connective tissue cells [229,230,231,232], MSCs [233,234,235], iPSCs [236,237], organoids [238,239,240,241,242], and connective tissue explants [243,244] have all have been shown to respond to mechanical loading, and in the case of meniscal explants [244] and tendon explants [245], they were shown to require in vitro mechanical loading to maintain tissue integrity.

Clearly, the potential role of EVs in tissue repair and regeneration has emerged as an important mechanism that is used by both MSCs and iPSCs [246,247,248,249,250]. Therefore, how the mechanical loading of the relevant cells influences EV release from these cells and potentially influences the cargo of EVs is not well understood. Furthermore, how the loading of tissues in vivo affects EV uptake is not well characterized. This area needs more investigation. As the loading of relevant cells in vitro can alter their responses to inflammatory cytokines such as IL-1 [251,252,253,254,255] and the in vivo environment [240], there is likely potential for mechanical loading to influence these other aspects of cell function both in vivo and in vitro. These topics should be the subject of future investigation.

Therefore, to optimize the translation potential of future investigation of the application of MSCs, iPSCs, and EVs, in vitro studies should be performed using conditions that best mimic the in vivo environment. For example, appropriate large animal models should that offer the best opportunities for translation should be employed, and injury and disease models should reflect those occurring in humans as accurately as possible. As the ability to respond to mechanical loading is critical for the in vivo function of repair tissue, assessing how such cells and constructs respond to appropriate mechanical loading in vitro may not only prepare the materials to better survive in the post-implantation environment, but may also enhance our understanding of how they function in such environments. For example, do cells generate appropriate EVs when they are loaded vs unloaded, and do EVs function in vivo under loading conditions the same as in vitro? The answers to such questions are likely important to foster in vivo post-implantation success and outcomes.

## 6. Summary and Conclusions

The effective cellular therapy for the repair and regeneration of connective tissues requires both the appropriate cells (i.e., MSC, iPSC) and cellular constituents (i.e., EVs), as well as an optimal environment to receive the interventions. Without congruence between these two factors, successful outcomes may be variable and thus impact the quality of life of the recipients. With regard to the cellular and EV components, there has to be a match between the application (i.e., treating an acute injury vs a chronic degenerative condition) and the source and characteristics of the cells and the cargo of the EVs. Although the emerging literature appears to indicate that the generation of EVs is an important mechanism by which MSCs, as regulatory cells, facilitate tissue repair, what the role of the “stemness” characteristics of these cells in vivo remains to be fully characterized and should be the focus of further study. Perhaps the “stemness” allows for the lineage-specific generation of EVs with specialized cargo to target endogenous cells in unique environments, as indicated by the results of Winston et al. [113].

With respect to the implantation environment, the inflammation and inflammatory background of the environment are likely a critical element that needs to be regulated. In the case of chronic degenerative conditions such as osteoarthritis, based on the findings indicating that the implanted materials appear to activate endogenous cells to facilitate repair [73], there needs to be sufficient endogenous tissue available to accomplish the repair goal. This means that repair using cellular therapy should be considered early after diagnosis rather than after the degeneration is very advanced. This scenario of an optimal cellular therapeutic vehicle coupled with an optimal implantation environment likely means that effective treatment will become personalized, and that the outcome expectations will be managed at the individual level. Furthermore, the presence of comorbidities and conditions that can influence outcomes should be considered when attempting to optimize the implantation environment. Continued progress towards achieving this dual goal should lead to enhanced outcomes going forward.

## Figures and Tables

**Table 1:** Multipotent stem cells (MSCs, iPSCs) and derivatives (EVs) for implantation and tissue repair: advantages and limitations.

	Advantages	Limitations
*Mesenchymal Stem/Stromal/Signaling Cells (MSC)*	Multiple tissue sources with different characteristics and potential lineages Can be obtained from some sources using minimally invasive methods (adipose tissue, bone marrow) or otherwise discarded source (placenta, cord blood, Wharton’s jelly) Readily cultured/expanded Can be used autologously Can be used to generate EVs containing unique cargo with or without prior differentiation Continuous generation of EVs in vivo after implantation Could be obtained from young adults and stored until needed	Numbers decrease with age in some sources Heterogeneity induced by extensive culturing and passaging Subsets of MSC may exist and remain to be isolated and optimized-different functions?
*IPSC*	Can be induced from specific somatic cells (i.e., skin or other tissue fibroblasts) Can be readily cultured and differentiated to specific lineages Can be used autologously Can be used to generate EVs with specific cargo ± lineage-specific differentiation Continuous generation of EVs in vivo after implantation Could be generated using young cells and then stored until needed	Initial cancer risk but risk decreasing with continued method alterations Heterogeneity resulting from continued expansion and instability?
*Extracellular Vesicles*	None of the risks associated with use of cells (i.e., DNA, cancer) Can be used as either an autologous or allogeneic intervention Cargo can be tailored to fit the application (from MSC or iPSC)	“Single use” approach and would require multiple implantations if continuous exposure required to optimize endogenous cell activation to facilitate repair Still requires further optimization of “targeting” endogenous cells to enhance repair function Additional research required to optimize cargo for specific applications Additional research required to further understand “mechanisms of action” in endogenous target cells to facilitate repair in specific applications

**Table 2:** Endogenous environmental factors that potentially influencing the success of cellular and EV therapies to improve connective tissue repair.

*Inflammation*	Acute inflammation associated with tissue injury Chronic inflammation associated with induction of failed repair and compromised healing, potentially due to endogenous cell impaired function
*Sufficient endogenous cells capable of responding to cellular and EV therapies*	Initiating interventions early before the cellular environment is devoid of required cells Controlling inflammation so endogenous cells can respond to interventions appropriately Secondarily, there remains sufficient extracellular matrix to serve as a template for repair tissue
*Co-Morbidities*	Diabetes-compromised healing a challenge to overcome Cardiovascular Disease-insufficient oxygenation and nutrients Obesity-metabolic syndrome, low grade systemic inflammation Post-menopausal Conditions-compromised bone integrity, compromised wound healing, etc.
*Other Variables*	Smoking Genetics/Epigenetics Consequences of Malnutrition
